# Wandering spleen with torsion causing an acute abdomen: A case report

**DOI:** 10.1016/j.ijscr.2024.110678

**Published:** 2024-11-29

**Authors:** Alazar Berhe Aregawi, Teketel Tadesse Geremew

**Affiliations:** a Department of Surgery, Hawassa University Comprehensive Specialized Hospital, Hawassa, Sidama, Ethiopia; b Department of Pathology, Hawassa University Comprehensive Specialized Hospital, Hawassa, Sidama, Ethiopia

**Keywords:** Splenectomy, Splenic torsion, Wandering spleen, Infarction, Case report

## Abstract

**Introduction and importance:**

Wandering spleen, also known as ectopic spleen, is an uncommon disorder in which the spleen's anatomical location differs from its fixed position in the abdomen's left upper quadrant. The etiology is either congenital or acquired, possibly leading to torsion and splenic infarction. It affects children and young adults, especially childbearing-age women. Patients affected by this condition may present with nonspecific symptoms requiring a high index of suspicion. Given the nonspecific clinical symptoms and the potential complications associated with wandering spleen, computed tomography scans provide a crucial means for proper diagnosis.

**Case presentation:**

A 38-year-old female patient presented with worsening abdominal pain of one-week duration. The pain was more localized to the left hemi abdomen but later she claimed that it became diffuse. She had associated vomiting of ingested matter and loss of appetite. She had a similar complaint of abdominal pain for the last year. Up on examination, she looked acutely sick. Abdominal examination showed a flat abdomen moved with respiration; a big intra-abdominal mass was tender; it was freely mobile in all directions; with no sign of fluid collection. A CT scan of the abdomen suggested an ectopic spleen with splenic torsion. Intraoperative findings revealed an infarcted wandering spleen. An emergency splenectomy was performed. The patient was discharged on the third postoperative day and had an uneventful postoperative recovery.

**Clinical discussion:**

If a normal spleen is not identified in the left upper quadrant, a search for ectopic splenic tissue should ensue. If the patient has not had a prior surgical splenectomy, some possible explanations include an ectopic or “wandering” spleen. This case was an infarcted wandering spleen caused by abnormal ligamentous attachments.

**Conclusion:**

Wandering spleen with torsion poses a great diagnostic challenge for acute abdomen due to the rarity of its occurrence and non-specific presentations. A high index of suspicion is the key to early diagnosis and timely intervention is required to improve treatment outcomes.

## Highlights

• Wandering spleen, also known as ectopic spleen, is an uncommon disorder in which the spleen's anatomical location differs from its fixed position in the abdomen's left upper quadrant.
• Wandering spleen with torsion poses a great diagnostic challenge for acute abdomen due to the rarity of its occurrence and non-specific presentations.
• Splenopexy and splenectomy are two alternatives for treatment in these patients, and the preferred alternative depends on the patient’s age, vascular status, and size of the spleen

## 1 Introduction

A wandering spleen is a rare clinical entity where the spleen is found abnormally somewhere else in the abdominal cavity or pelvic cavity instead of its normal anatomical position in the left hypochondrium [1]. It is a rare clinical entity with a <0.2 % reporting incidence rate, and the spleen can be found in several positions in the abdomen or pelvis, and this condition is a result of congenital malformation or agenesis of the splenic ligaments or ligamentous laxity due to trauma, pregnancy, and connective tissue diseases [2]. Clinical presentation ranges from asymptomatic abdominal mass to intestinal obstruction or acute abdominal, which requires urgent surgical intervention. Its diagnosis is incidental in asymptomatic patients when a patient needs investigation for any other medical problem. In symptomatic cases, it may be diagnosed with splenic torsion, infraction, and splenic rupture, which can lead to an acute abdomen [2,3]. Imaging studies have an important role in diagnosis, and computed tomography and ultrasound are the preferred modalities; splenic angiograms are very helpful in diagnosis [[4], [5], [6], [7]]. Splenopexy and splenectomy are two alternatives for treatment in these patients, and the preferred alternative depends on the patient's age, vascular status, and size of the spleen [[7], [8], [9], [10]]. We present a rare case of wandering spleen with torsion in a thirty-eight-year-old female patient who presented with an acute abdomen, and an emergency splenectomy was performed. From our literature search, there are very few reported cases of wandering spleen from Ethiopia, and there are only a few cases reported in the rest of the world. This case report is reported in line with SCARE criteria [11].

## 2 Case

Our case is a 55-year-old female patient from Ethiopia, who presented with worsening abdominal pain of one-week duration. The pain was more localized to the left hemi abdomen but later she claimed that the pain became diffuse. She had associated vomiting of ingested matter, about four episodes, and loss of appetite. She had a similar complaint of intermittent abdominal pain for the last year. She is a para V mother.

She has no history of trauma to the abdomen.

She has no history of immunocompromization.

She has no family history of similar illness.

Physical examination, General appearance: She looked acutely sick. Vital signs: pulse rate 120 beats per minute, respiratory rate- 22 breaths per minute, Temperature: 36.3, blood pressure: 107/79 mmHg.

**Fig. 1:**
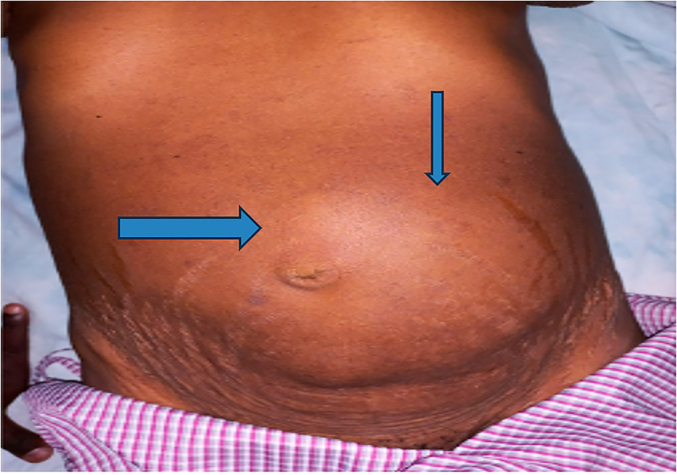
A big intra-abdominal mass about 10 × 8 cm in size and tender to touch; it was freely mobile in all directions.

Abdomen-flat moved with respiration; there was a big intra-abdominal mass located more on the left lower quadrant that was about 10 × 8 cm in size and tender to touch; it was freely mobile in all directions, with no sign of fluid collection (Fig. 1).

## 3 On investigation

**Fig. 2:**
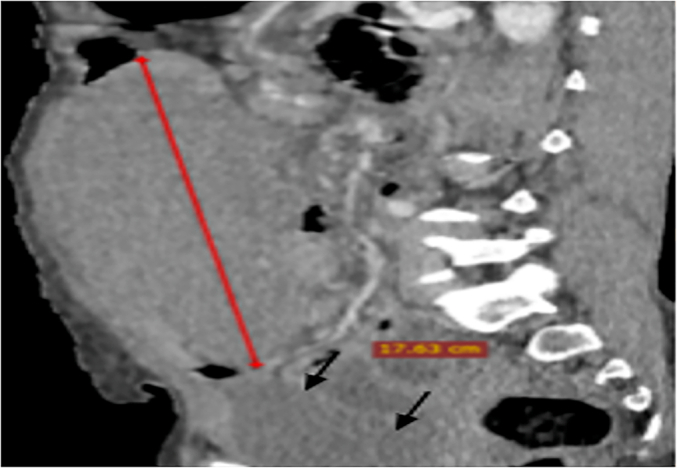
Lower pelvic mass representing the mal-positioned spleen, and homogenous splenic parenchyma with loss of attenuation.

**Fig. 3:**
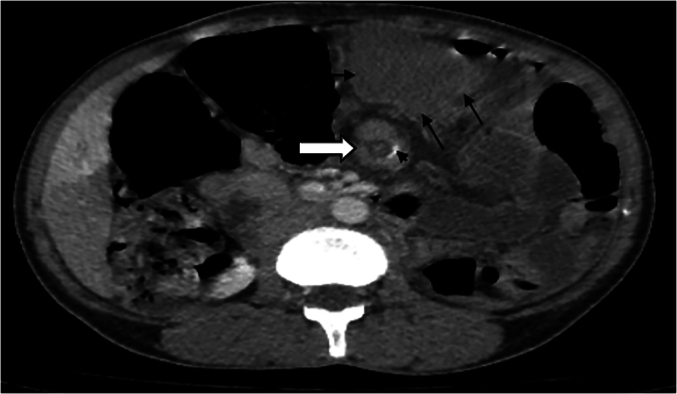
Whirl sign as a specific sign for splenic torsion in wandering spleen.

Complete blood count (white cell count = 15.2/L Neutrophils = 12.4 % Lymphocytes = 1.6 % Eosinophils = 1.2 %, Hgb = 12.3 g/d, Platelet = 472 × 10^3^/L), Blood group & Rh = O+, liver and renal function tests were within the normal range. Abdominal ultrasound, the spleen was not visualised in its normal left upper quadrant location. A contrast enhanced CT scan of the abdomen which showed a wandering spleen with torsion (Fig. 2, Fig. 3).

**Fig. 4:**
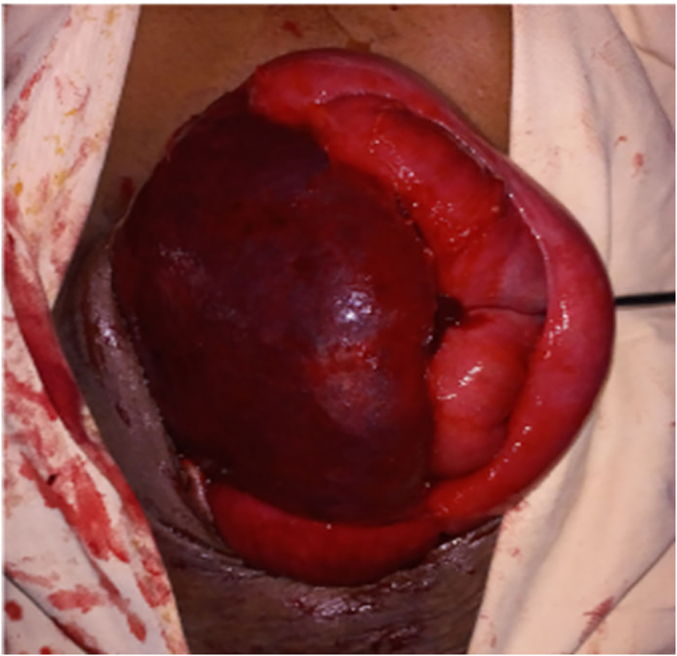
Tensioned spleen with no attachment to the diaphragm or anterolateral wall.

**Fig. 5:**
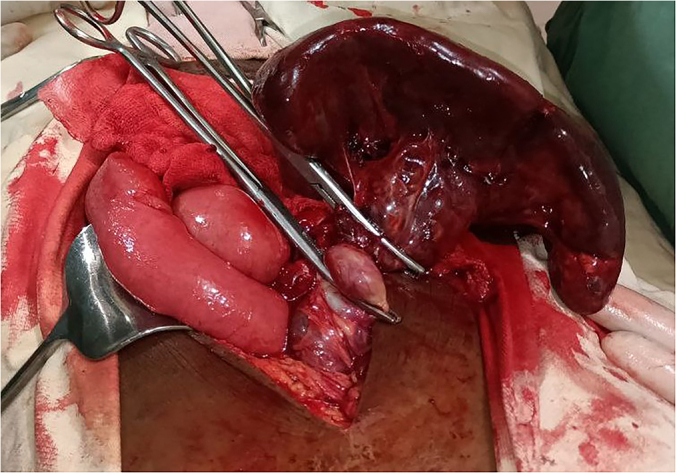
Ischemic spleen with no attachment.

**Fig. 6:**
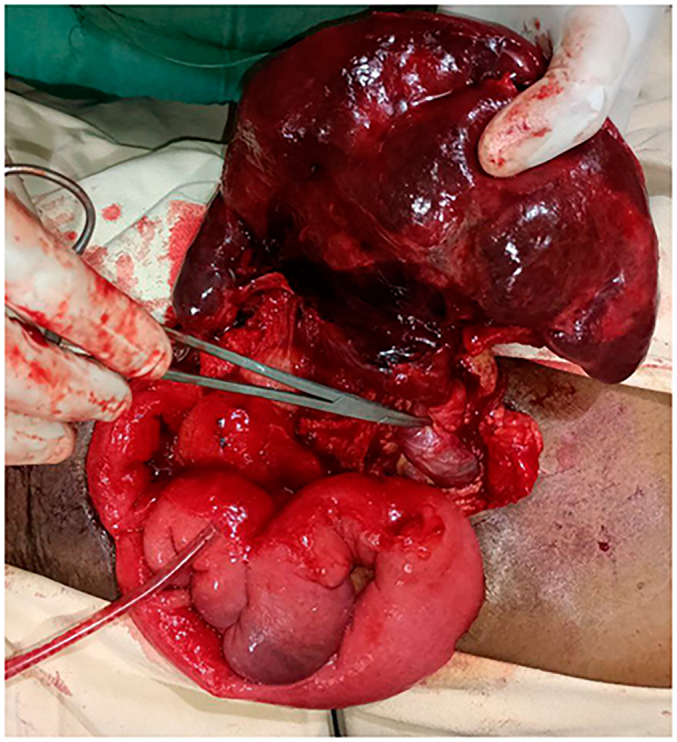
Ischemic spleen with ecchymotic outer surface.

Considering the findings, exploratory laparotomy was decided. We put her on maintenance fluid with N/S, kept her NPO, and took her to the operation theatre after a few hours. We explored the abdomen, and we did an exploratory laparotomy. Intraoperative findings: the spleen was hugely enlarged, extending to the pelvis. It was freely mobile. The pedicle has torsed and part of the vessels were thrombosed. The spleen was ischaemic, and then we clamped the pedicle and did a splenectomy without detorsing the vessels. Upon exploration, we did not identify other splenic tissue at the splenic bed or elsewhere. We closed the abdomen after thorough lavage and the count was declared correct (Fig. 4, Fig. 5, Fig. 6).

The patient had a smooth post-op course and was discharged on the 3rd post-op day with vaccination given on the 2nd week and she was evaluated at the 3rd week with good post-OP condition.

## 4 Discussion

The spleen is normally positioned in the left upper quadrant and fixed into position by the splenorenal, spleno-colic, and gastrosplenic ligaments [12]. Wandering spleen, also known as floating spleen, ectopic spleen, ptotic spleen, or splenoptosis, can move anywhere in the abdominal or pelvic cavity, attached only by its vascular pedicle and unattached to the surrounding structures. The long pedicle allows it to torsion on itself, which can lead to complications. The degree of torsion, according to literature, ranges from 90° to 2160o [10,13]. Van Horne introduced the concept of wandering spleen in 1667 [14]. In 1854, a Polish physician named Jozef Dietl published one of the first documented cases of a child's wandering spleen [15]. It is a rare entity with a reported incidence rate of <0.2 %, accounting for 0.002 % (two per thousand) splenectomies [16]. Till now, <700 cases of wandering spleen have been reported in literature ranging from 3 months to 82 years [10]. From our literature search, <5 cases are reported in Ethiopia. Ectopic spleens can be either congenital or acquired. Congenital causes include developmental anomalies in the dorsal mesogastrium, which fails to fuse with the posterior peritoneum during the fifth and sixth weeks of foetal development, resulting in the absence or abnormal development of one or more suspensory splenic ligaments (gastrosplenic, phrenocolic, and splenorenal). Connective tissue abnormalities, multiparity, hormonal changes, splenomegaly, trauma, and abdominal wall weakening are considered acquired causes of ligament laxity [10,17,18]. Our patient is multiparous with Para IV.

Up to one-third of all cases appear to be children, with 30 % being under 10 years old. The male-to-female ratio is 1:1 in this population. The second peak in incidence occurs between 20 and 40 years, with the male-to-female ratio changing to 1:7, potentially further enhancing the association of wandering spleen with pregnancy [19]. The increased occurrence among women of childbearing age is connected with hormonal changes, multiple pregnancies, and abdominal wall weakening [10].

It can be asymptomatic and discovered only on imaging or during clinical examination as a palpable mass and may never be symptomatic [20]. Another set of patients might present with recurrent attacks of abdominal pain due to torsion and spontaneous detorsion of the splenic pedicle. Our patient falls under this category of presentation. The acute surgical abdomen is a more serious presentation secondary to splenic torsion and subsequently acute splenic infarction, a life-threatening condition [21].

Diagnostic modalities, including CT, magnetic resonance imaging, or Doppler ultrasound, can help to confirm the diagnosis; however, a CT scan remains the investigation of choice not only to confirm the diagnosis of WS but more importantly to rule out other causes of acute abdomen as this condition can mimic other pathologies, especially in women of childbearing age where it can present as ovarian torsion or rupture ectopic pregnancy [22]. An abdominal US and CT scan was done for our patient.

Findings on the CT scan include the absence of the spleen in its normal position, lower pelvic mass representing the malpositioned spleen, and homogenous or heterogeneous splenic parenchyma with loss of attenuation [12]. This finding was similar to our case.

Surgery is the gold standard treatment of wandering or ectopic spleen. Splenopexy and splenectomy are two procedures available for surgical management of WS. Surgical procedure depends on the condition of the wandering spleen, especially on the viability of the spleen. Laparoscopic or open splenopexy for uncomplicated cases of WS. Various methods of splenopexy, such as suturing the capsule of the spleen to the left upper quadrant of the abdomen and forming an extraperitoneal pocket for the spleen at the 12th rib level and suturing the greater curvature of the stomach to the anterior abdominal wall, are available [23,24]. In recent improvements, polyglycolic mesh as a ‘snood’ has been utilized to anchor the spleen, and the sandwich approach, in which two meshes are used to sandwich the spleen, is reported in the literature for laparoscopic splenopexy [25]. When the spleen is enlarged, infarcted, ruptured, or there are indicators of hypersplenism, the choice of therapy is splenectomy, in which the spleen is completely removed either laparoscopically or through laparotomy [10]. In our patient, a splenectomy was done due to splenic torsion with thrombosed vessels and an infarcted spleen. Post splenectomy, all patients should receive pneumococcus, meningococcus, and *Hemophilus influenza* vaccines [26]. We have advised our patient to take the vaccines two weeks post-op and she should take booster doses subsequently.

## 5 Conclusion

The wandering spleen is a comparatively rare clinical entity, that can present with various signs and symptoms. Emergency room physicians and surgeons should be aware of this entity, especially when the patient presents with an acute abdomen. Timely diagnosis and interventions are crucial for spleen preservation and avoiding life-threatening complications. Surgery for splenopexy or splenectomy is the treatment of choice depending on the condition of the spleen.

## Abbreviations

CT: computed tomography
US: ultrasound
WS: wandering spleen

## Author contribution

Alazar Berhe Aregawi, MD: Involved in acquisition of data, literature review of the paper, writing and drafting the paper, editing and critical review of the paper.

Teketel Tadesse Geremew, MD: literature review of the paper, writing and drafting the paper, editing and critical review of the paper.

## Patient consent

The report was done with patient's consent.

## Consent

Written informed consent was obtained from the patient, by their native language, for publication of non-identifying information including accompanying intraoperative images. A copy of the written consent is available for review by the Editor-in-Chief of this journal on request.

## Ethical approval

Written informed consent was obtained from the patient for publication and any accompanying images. A copy of the written consent is available for review by the Editor-in-Chief of this journal on request.

## Guarantor

Teketel Tadesse Geremew, MD.

## Funding

None.

## Conflict of interest statement

The authors declare that there are no conflicts of interest on this case report.
